# Sodium Butyrate Promotes In Vitro Development of Mouse Preantral Follicles and Improves Oocyte Quality by Regulating Steroidogenesis, Oxidative Stress, and Cytoskeleton Remodeling

**DOI:** 10.3390/ani15243567

**Published:** 2025-12-11

**Authors:** Xiaohuan Liu, Tuba Latif Virk, Mengdie Pi, Qi Liu, Sheng Yang, Zhiyu Ma, Yuguo Yuan, Fenglei Chen

**Affiliations:** 1 College of Veterinary Medicine, Yangzhou University, Yangzhou 225009, China; lxh136288@163.com (X.L.); tuba.virk2@gmail.com (T.L.V.); pp010177@163.com (M.P.); qiliu@yzu.edu.cn (Q.L.); 008220@yzu.edu.cn (S.Y.); mzy2017@yzu.edu.cn (Z.M.); yyg9776430@163.com (Y.Y.); 2 Jiangsu Co-Innovation Center for Prevention and Control of Important Animal Infectious Diseases and Zoonoses, Yangzhou 225009, China; 3 Joint International Research Laboratory of Agriculture and Agri-Product Safety of the Ministry of Education of China, Yangzhou University, Yangzhou 225009, China

**Keywords:** sodium butyrate, preantral follicle, oocyte, oxidative stress, mouse

## Abstract

Sodium butyrate (NaBu), a common feed additive, has been shown to enhance reproductive performance in livestock and poultry. However, whether NaBu exerts this effect by directly regulating follicular development remains unclear. In this study, a three-dimensional (3D) in vitro culture system of mouse preantral follicles was used to investigate the effects of NaBu on follicular growth, hormone secretion, maturation of oocytes, and subsequent embryonic development. Preantral follicles were treated with different doses of NaBu on the fourth day of culture. Subsequently, the mature oocytes (MII stage) were released from the follicles on the ninth day and subjected to parthenogenetic activation for developmental assessment. The results showed that 0.10 mM NaBu treatment could significantly promote follicular growth, antral formation, and oocyte maturation. Furthermore, NaBu also significantly increased estradiol (E_2_) secretion, improved follicular structure, and maintained cellular viability. qPCR analysis revealed that NaBu significantly increased the mRNA levels of *STAR*, *CYP11A1*, and *CYP1B1*. In addition, it significantly enhanced the distribution and organization of F-actin, with increases in the mRNA levels of *GDF9*, *BMP15*, and *CX37*. NaBu treatment significantly reduced intracellular ROS levels and increased the mRNA levels of *NRF2* and *SOD1*, while *SOD2* and *GSR* showed increasing trends without significant differences. NaBu significantly improved oocyte cytoskeletal organization and the morphology of the spindle, but it did not lead to a significant increase in the rates of cleavage and blastocyst formation after parthenogenetic activation. Collectively, these findings indicate that NaBu promotes follicular development and improves oocyte quality, at least partly, by enhancing steroidogenesis, alleviating oxidative stress, and maintaining cytoskeletal integrity, providing insight into its potential application for improving reproductive performance in livestock and poultry.

## Simple Summary

Sodium butyrate (NaBu) is a natural compound that is commonly used as a feed additive to improve the growth and health of animals, but its direct influence on follicular development has not been clearly understood. In this study, a three-dimensional (3D) culture system of mouse follicles was used to examine the effects of NaBu on follicular growth, hormone secretion, and the development of oocytes. Our results showed that NaBu could promote follicular growth and maturation, increase estrogen secretion, maintain healthy follicular structure, and enhance oocyte quality by improving cytoskeletal organization and reducing oxidative stress. It also supported communications between oocytes and ovarian granulosa cells, contributing to better follicular function. Although early embryonic development after fertilization did not show a significant difference, NaBu effectively improved the overall developmental potential of the follicles and oocytes. These findings suggest that NaBu has significant potential as a regulatory factor for optimizing in vitro follicular culture systems and improving the quality of oocytes, with broad application prospects in animal reproduction and assisted reproductive technologies.

## 1. Introduction

Feed additives are effective nutritional interventions that have been widely applied to improve reproductive performance in livestock and poultry [1]. Reproductive efficiency in livestock and poultry largely depends on ovarian development, which ensures the growth of healthy follicles, maturation of oocytes, and secretion of reproductive hormones that are necessary for ovulation, fertilization, embryonic development, and the production of healthy offspring [2].

Sodium butyrate (NaBu), a short-chain fatty acid produced by the microbial fermentation of dietary fiber in the gut of ruminants, has received widespread attention for its diverse biological functions, including the regulation of energy metabolism, maintenance of intestinal homeostasis, and modulation of immune responses [3]. As an important feed additive, NaBu plays essential roles in improving the intestinal microenvironment, enhancing immune function, and promoting growth [4,5]. In addition, its positive effects on reproductive performance in livestock and poultry have been demonstrated. Supplementation of NaBu in the diet of sows can enhance immune status, reduce inflammation, and increase the survival rates of embryos and newborn piglets [6,7]. In poultry, NaBu enhances the deposition of antioxidant substances in egg yolks, thereby improving the antioxidant capacity of embryos, enhancing embryonic quality, and increasing chick survival rates [8].

Beyond these systemic effects, NaBu has also been reported to influence embryonic development at the cellular level. Studies in mammalian oocytes and embryos have reported that NaBu can enhance mitochondrial activity, support ATP production, reduce intracellular reactive oxygen species (ROS), stabilize cytoskeletal organization, and improve overall cellular homeostasis by modulating metabolic pathways and strengthening antioxidant defenses [9]. It has also been implicated in regulating steroidogenesis in ovarian granulosa cells, including modulation of key genes that are involved in estradiol and progesterone synthesis [10]. These findings collectively suggest that NaBu may directly affect ovarian granulosa cells and the oocytes in the follicular environment. However, whether the effects of these cellular and endocrine regulatory occur in the intact follicular structure, particularly during the preantral to antral transition, remains to be clarified.

Although these reproductive benefits of NaBu have been reported, its direct effects on ovarian follicles remain unclear. Mouse follicles are commonly used as an experimental model, because their developmental patterns share several features with those of other mammals, and because the in vitro culture system is relatively well-established [11]. This model allows for controlled observation of the follicular growth and maturation of oocytes, providing a practical approach for exploring cellular responses to NaBu.

However, no systematic studies have examined the effects of NaBu on the complete transition from preantral to mature follicles [10,12]. Therefore, based on these observations, we hypothesized that NaBu might promote follicular development and improve the quality of oocytes by modulating steroidogenesis, reducing oxidative stress, and supporting cytoskeletal organization in the follicular microenvironment. Accordingly, the current study was designed to evaluate the effects of NaBu on follicular development and elucidate the underlying mechanisms using a three-dimensional (3D) in vitro culture system of mouse preantral follicles.

## 2. Materials and Methods

### 2.1. Collection of Mouse Preantral Follicles

Female ICR mice (12.5 days old) were obtained from the Laboratory Animal Center of Yangzhou University. All procedures were approved by the Animal Protection and Ethics Committee of Yangzhou University (Approval No. 202103322). In each replicate, 3 mice were used, yielding 6 ovaries and about 96 preantral follicles. Three independent biological replicates were performed in this study, while 9 12.5-day-old female mice were used. The mice were euthanized by CO_2_ overdose, and their ovaries were collected under aseptic conditions. After removing oviducts and surrounding fat, the ovaries were rinsed in L-15 medium containing 10% fetal bovine serum (FBS; Sigma-Aldrich, St. Louis, MO, USA; Cat. No. F8318-500 mL) and 1% penicillin–streptomycin (Thermo Fisher, Waltham, MA, USA; Cat. No. 15140-122). Preantral follicles (100–130 μm, 2–4 granulosa cell layers, and intact basal membrane) were isolated using a 1 mL syringe with a 0.3 × 13 RWLB needle under a stereomicroscope and selected for subsequent culture.

The use of 12.5-day-old prepubertal mice is consistent with established in vitro follicular culture protocols, as their ovaries contain a high proportion of uniformly staged preantral follicles and provide a reproducible model for studying folliculogenesis [13]. Unlike adult cycling mice, in which follicular heterogeneity increases due to ongoing recruitment and atresia, prepubertal ovaries offer more synchronized follicular populations, thereby improving consistency and experimental control.

### 2.2. Preantral Follicles for 3D In Vitro Culture

Preantral follicles were cultured in ultra-low-attachment 96-well plates (Corning, NY, USA; Cat. No. 4520) coated with a hydrogel layer that minimizes cell adhesion and prevents ovarian granulosa cells from attaching to the well bottom [13].

Each well was filled with 200 μL of follicular growth medium consisting of Minimum Essential Medium alpha (MEM-α; Thermo Fisher, Waltham, MA, USA; Cat. No. C12571500BT) supplemented with 0.33 mM sodium pyruvate (Sigma-Aldrich, St. Louis, MO, USA; Cat. No. P5280-25G), 10% FBS (Sigma-Aldrich, St. Louis, MO, USA; Cat. No. F8318-500 mL), 1% penicillin–streptomycin (Thermo Fisher, Waltham, MA, USA; Cat. No. 15140-122), 1% insulin–transferrin–selenium (ITS; Thermo Fisher, Waltham, MA, USA; Cat. No. 41400045), 0.1 IU/mL human chorionic gonadotropin (HCG; Ningbo Second Hormone Factory, Ningbo, China; Cat. No. 10-1255), and 50 μg/mL vitamin C (Solarbio Science & Technology Co., Beijing, China; Cat. No. 02-1178). The plates were pre-equilibrated at 37.5 °C in a humidified atmosphere containing 5% CO_2_ for 2 h before use. Individual preantral follicles were then placed into separate wells and cultured for 8 consecutive days, with the first 24 h being defined as day 1. Half of the culture medium was replaced with fresh medium every 2 d, and the spent medium was collected and stored at –80 °C for subsequent estradiol (E_2_) analysis.

On the fourth day, the follicles were cultured in the medium containing 0.10 mM NaBu (Sigma-Aldrich, St. Louis, MO, USA; Cat. No. B5887-250 mg) for 2 d. This dose was selected based on preliminary experiments, in which 0.10 mM showed the most stable follicular response among several doses (Supplementary Materials). Day 4 was chosen because the follicles at this stage had formed early antral structures and showed a more consistent response to external factors than in earlier stages. On the sixth day, the medium was completely replaced with NaBu-free medium to terminate the treatment. Follicular growth was monitored daily under an inverted microscope (Olympus CKX53, Tokyo, Japan). On the eighth day, the follicle growth medium was replaced with oocyte mature medium, composed of follicular growth medium supplemented with 1.5 IU/mL HCG (Ningbo Second Hormone Factory, Ningbo, China; Cat. No. 10-1255) and 10 ng/mL epidermal growth factor (EGF; PeproTech, Cranbury, NJ, USA; Cat. No. AF-100-15-100 μg). Cumulus–oocyte complexes (COCs) were observed to ovulate spontaneously. After 14–16 h, the rate of ovulation was recorded on the ninth day.

### 2.3. Oocyte Collection and Parthenogenetic Activation

After 14–16 h of oocyte maturation medium addition, COCs were collected and examined for ovulation. The presence of extruded first polar bodies was used to confirm the presence of oocytes at the metaphase II (MII) stage. Cumulus cells were removed by treating the COCs with 0.1% hyaluronidase at 37 °C for approximately 2 min, followed by gentle pipetting.

The oocytes were transferred into a calcium-free Chatot–Ziomek–Bavister (CZB) medium containing strontium chloride (SrCl_2_), which had been pre-equilibrated at 37 °C with 5% CO_2_ for 1 h. Parthenogenetic activation was performed for 20 min, following the method described by Li et al. [14].

After activation, the oocytes were washed three times with potassium simplex optimization medium (KSOM) and then transferred into fresh embryonic culture medium (2–4 embryos/μL) for further development. The embryonic culture medium used in this study was supplied as a commercial formulation by Jiangsu Jicui Yaokang Biotechnology Co., Ltd. (Nanjing, China). According to the manufacturer, it is intended for mouse embryonic development and functionally resembles KSOM-based medium, although the detailed compositions were not disclosed. Embryonic morphology was observed at 24, 48, 72, and 96 h under an inverted microscope (Olympus CKX53, Tokyo, Japan). The activation of the oocytes was evaluated after 6 h of parthenogenetic activation. Oocytes exhibiting extrusion of the second polar body, along with the formation of pronuclei, were counted as activated oocytes, and the rate of activation was calculated accordingly. The rate of cleavage was assessed after 24 h of activation by counting embryos that had developed to the 2- or 4-cell stage. The rate of blastocyst formation was evaluated at 96–120 h by determining the number of embryos that had reached the blastocyst stage.

### 2.4. Measurement of E_2_ Secretion In Vitro

At each medium change on the second, fourth, sixth, and eighth days, 100 μL of spent culture medium was collected and pooled into one sample for every 10 follicles. All samples were immediately frozen and stored at −80 °C until use.

The concentrations of E_2_ in the spent medium were determined using a mouse-specific enzyme-linked immunosorbent assay (ELISA) kit (FanKoway Biotech, Nanjing, China; Cat. No. F2546-A) according to the manufacturer’s instructions. The assay exhibited an intra-assay coefficient of variation (CV) < 8% and an inter-assay CV < 10%, with a detection range of 5–2000 pg/mL. The absorbance was measured at 450 nm using a microplate reader (Bio-Rad, Hercules, CA, USA), and the concentrations of E_2_ were calculated from a standard curve generated using serial dilutions of the E_2_ standard.

### 2.5. Observation of Follicular Morphology and Measurement of Follicular Diameter

On the second, fourth, sixth, and eighth days, follicular morphology, survival, diameter, and antral formation were observed and recorded under an inverted microscope (Olympus CKX53, Tokyo, Japan). The follicular diameter was measured using ImageJ 1.54 (NIH, Bethesda, MD, USA) by calculating the mean of the longest and perpendicular diameters.

### 2.6. Evaluation of Follicular Survival, Antral Formation, and Ovulation Rates

Follicular survival was assessed based on morphological integrity and transparency. Follicles that remained intact, spherical, and transparent, with no detachment of ovarian granulosa cells from the basal membrane, were classified as survival follicles. The rate of antral formation was determined by the appearance of a visible antrum in the follicle and calculated as the percentage of the follicles that developed a clear antrum among all cultured follicles. The rate of ovulation was assessed after 14–16 h of oocyte mature medium addition by observing the release of oocytes and COCs and was calculated as the percentage of the follicles that ovulated relative to the total number of all cultured follicles. The rates of follicular survival, antral formation, and ovulation were determined using an inverted microscope (Olympus CKX53, Tokyo, Japan).

### 2.7. Detection of Follicular Viability and F-Actin Cytoskeleton

After NaBu treatment, the follicles were collected on the eighth day. Fifteen follicles per replicate were used in each experiment, and three independent replicates were performed. The follicles were washed three times with calcium- and magnesium-free PBS. For viability assessment, the follicles were incubated with Calcein-AM (1:1000; GC Biotech, Waddinxveen, The Netherlands; Cat. No. GCA-100) at 37 °C for 30 min in the dark, washed with PBS three times, and then stained with propidium iodide (PI; Beyotime, Shanghai, China; Cat. No. ST511) for 5 min. For F-actin cytoskeleton staining, the follicles were fixed in 3.7% formaldehyde for 15 min, permeabilized with 0.1% Triton X-100 for 10 min, and incubated with Phalloidin-iFluor 488 (1:1000; Abcam, Cambridge, UK; Cat. No. ab176756) for 90 min. The nuclei were counterstained with DAPI (Beyotime, Shanghai, China; Cat. No. C1005) for 10 min. Fluorescence signals were visualized using a confocal laser scanning microscope (TCS SP8 STED, Leica, Wetzlar, Germany), and mean fluorescence intensity (MFI) was quantified using ImageJ (NIH, Bethesda, MD, USA).

### 2.8. Detection of Spindle Morphology and Mitochondrial and ROS Distribution in Oocytes

After NaBu treatment, denuded oocytes were collected by treating COCs with 0.1% hyaluronidase on the ninth day. Fifteen oocytes per replicate were included in each experiment, and three independent replicates were performed. For the morphology of the spindle, the oocytes were fixed with 3.7% formaldehyde for 15 min, stained with Tubulin-Tracker Green (1:1000; Beyotime, Shanghai, China; Cat. No. C1051S) for 30 min, and counterstained with DAPI (Beyotime, Shanghai, China; Cat. No. C1005) for 10 min. For mitochondrial distribution analysis, the oocytes were incubated with MitoTracker Red (1:1000; Beyotime, Shanghai, China; Cat. No. C1035) at 37 °C for 30 min, while for ROS detection, the oocytes were incubated with 10 μmol/L DCFH-DA (Beyotime, Shanghai, China; Cat. No. S0033S) at 37 °C for 20 min. All samples were imaged under an inverted microscope (Olympus IX73, Tokyo, Japan). Fluorescence intensity was analyzed using ImageJ (NIH, Bethesda, MD, USA).

### 2.9. RNA Extraction and Quantitative PCR (qPCR)

After NaBu treatment, 80 follicles were collected in each replicate on the 8th day and three independent replicates were performed. The total RNA of the follicles was extracted using TRIzol reagent (TaKaRa Bio, Dalian, China; Cat. No. 9109). The concentration and purity of RNA were assessed using a NanoDrop 2000 spectrophotometer (Thermo Fisher, Waltham, MA, USA). The samples with A260/A280 ratios of 1.8–2.2 were used for cDNA synthesis and qPCR. Reverse transcription was conducted using a commercial kit (TaKaRa Bio, Dalian, China; Cat. No. RR036A), and qPCR was performed with SYBR Green (TaKaRa Bio, Dalian, China; Cat. No. RR820A) on a Roche Light Cycler 480 II system (Roche Diagnostics, Indianapolis, IN, USA). Primer sequences are listed in Table 1. Gene expression was calculated using the 2^−ΔΔCt^ method and normalized to GAPDH.

### 2.10. Statistical Analysis

All results are presented as mean ± standard deviation (SD). Statistical analyses were performed using GraphPad Prism 9.0 (GraphPad Software, San Diego, CA, USA) and SPSS 26.0 (IBM Corp, Armonk, NY, USA). One-way ANOVA and Student’s *t*-test were used for comparison among groups, and a *p*-value < 0.05 was considered statistically significant.

## 3. Results

### 3.1. In Vitro Isolation of Mouse Preantral Follicles

**Figure 1:**
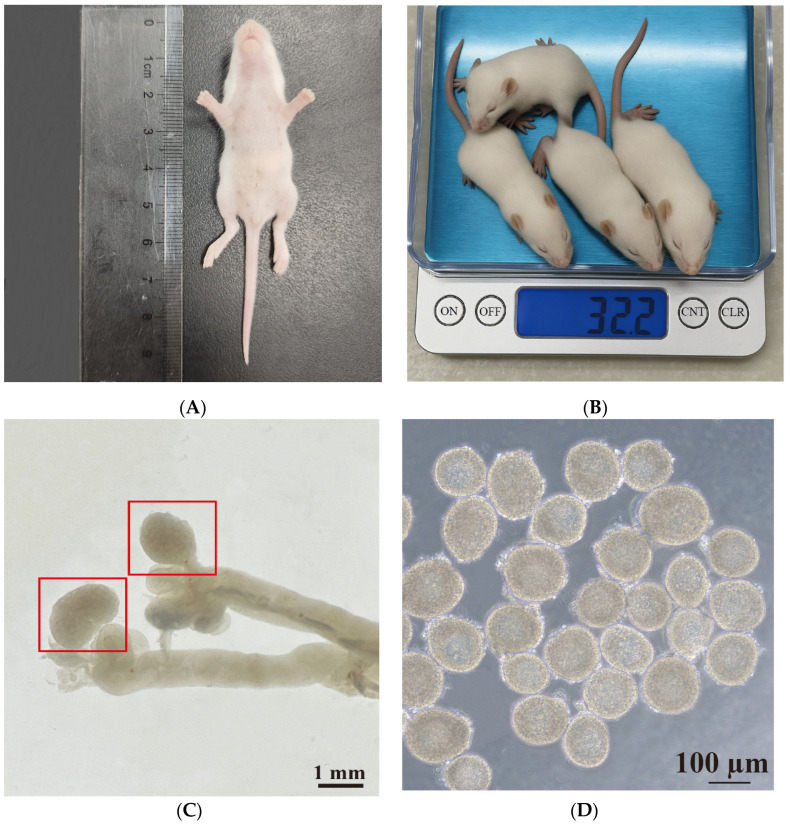
Isolation and characterization of preantral follicles from 12.5-day-old ICR mice. (**A**) Measurement of body length. (**B**) Assessment of body weight. (**C**) Representative image of ovaries isolated from 12.5-day-old female ICR mice. Red boxes represent the ovaries. (**D**) The morphology of typical preantral follicles. Scale bars, 1 mm (**C**) and 100 μm (**D**).

In this study, 12.5-day-old female ICR mice were used. The average body length of the mice was 6.00 cm ± 1.32 cm (Figure 1A), and the average body weight was 8.40 g ± 0.50 g (Figure 1B). During the procedure, both ovaries were excised and immediately placed in L-15 medium (Figure 1C). Preantral follicles with a diameter of 100–130 μm and intact basal membranes, surrounded by 2–4 layers of ovarian granulosa cells, and containing clearly visible oocytes were selected for further culture (Figure 1D).

### 3.2. In Vitro 3D Culture of Mouse Preantral Follicles

**Figure 2:**
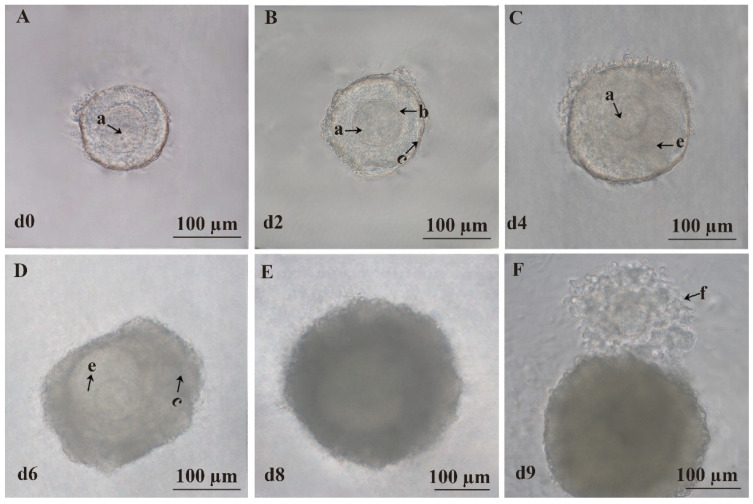
Three-dimensional culture of preantral follicles in vitro. (**A**) Representative image of the follicle on the zeroth day (d0). (**B**) Representative image of the follicle on the second day (d2). (**C**) Representative image of the follicle on the fourth day (d4). (**D**) Representative image of the follicle on the sixth day (d6). (**E**) Representative image of the follicle on the eighth day (d8). (**F**) Representative image of the follicle on the ninth day (d9). (a) Oocyte. (b) Zona pellucida. (c) Granulosa cells. (e) Follicular antrum. (f) COCs. Scale bar, 100 μm.

On the zeroth day of culture, the follicles were surrounded by 2–4 layers of ovarian granulosa cells with centrally located, morphologically intact, and nearly spherical oocytes (Figure 2A). On the second day, the follicles gradually increased in size with a slight increase in ovarian granulosa cell layers, while the size of the oocytes remained relatively unchanged (Figure 2B). On the fourth day, the oocytes began to shift from the center, and some follicles started to form an antrum (Figure 2C). On the sixth day, the antrum became more prominent, and ovarian granulosa cells proliferated significantly, forming multilayered dense structures (Figure 2D). On the eighth day, the diameter of the follicles continued to increase, and the antrum exhibited enhanced refractivity (Figure 2E). Meanwhile, oocyte mature medium was added, and after 14–16 h of further culture, ovulation was observed with typical COCs showing radiating cumulus cells surrounding the oocyte on the ninth day (Figure 2F).

### 3.3. Effects of NaBu on Follicle Morphology, Diameter, Survival, Antral Formation, and Ovulation In Vitro

On the zeroth day, NaBu was added to the culture medium at doses of 0.05, 0.10, 0.5, and 1.0 mM. There were no significant differences in follicular diameters among all the groups on the zeroth and second day (Table S1). Increases in follicular diameter were significantly inhibited in the 0.5 and 1.0 NaBu groups, while there were no significant difference in the 0.05 and 0.10 NaBu groups compared to the control group on the fourth, sixth, and eighth days (Table S1). Correspondingly, the rates of follicular survival, antral formation, and ovulation were significantly decreased in the NaBu group compared with the control group (Table S2).

**Figure 3:**
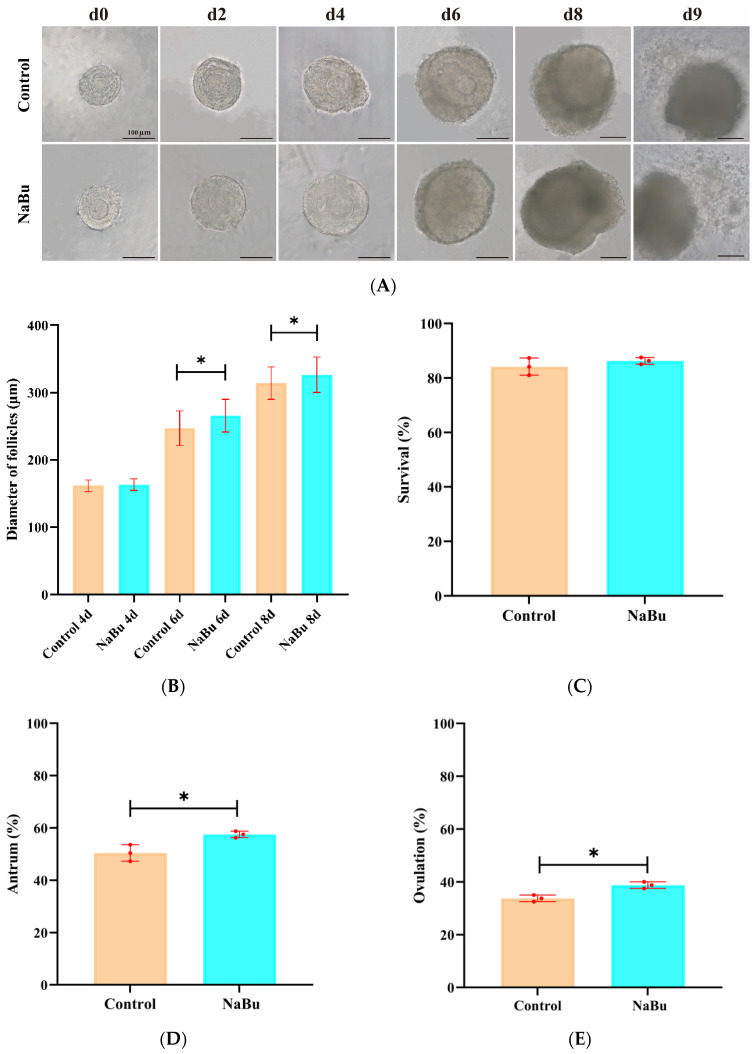
Effects of NaBu on follicular development when added on day 4 of culture in vitro. (**A**) Representative morphological images of the follicles on the eighth day. (**B**) Follicular diameter on the fourth, sixth, and eighth days. (**C**) Rate of follicular survival. (**D**) Rate of antral formation. (**E**) Rate of ovulation. Data represent mean ± SD from three independent experiments (*n* = 3), with 80 follicles being analyzed per group (*N* = 80). * *p* < 0.05 vs. Control. Scale bar, 100 μm.

After a 2-day 0.10 mM NaBu treatment starting on the fourth day, the follicles maintained good structural integrity, and displayed increased ovarian granulosa cell layers, a gradually developed antrum, and similar transparency levels compared with the control group on the eighth day (Figure 3A). After the addition of oocyte mature medium on the ninth day, both the control and NaBu groups showed successful ovulation and release of typical COCs (Figure 3A). Before NaBu treatment on the fourth day, there was no significant difference in follicular diameters between the NaBu and control groups. However, after NaBu supplementation, follicular diameters were significantly increased in the NaBu group on the sixth day and eighth days compared with the control group (Figure 3B). The rate of follicular survival exhibited no significant difference between the two groups (Figure 3C); while the rate of antral formation was significantly increased in the NaBu group compared with the control group (Figure 3D), as was the rate of ovulation (Figure 3E).

### 3.4. Effects of NaBu on E_2_ Secretion

**Figure 4:**
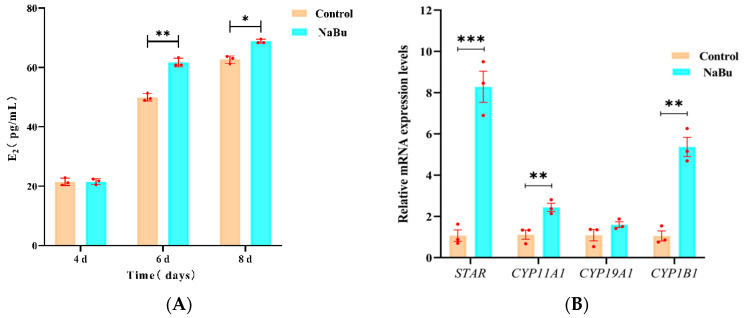
Effects of NaBu on E_2_ secretion when added on day 4 of culture in vitro. (**A**) Concentration of E_2_ in culture medium on the fourth, sixth, and eighth days. (**B**) Relative mRNA levels of *STAR*, *CYP11A1*, *CYP19A1*, and *CYP1B1*. Data represent mean ± SD from three independent experiments (*n* = 3), with 10 samples being analyzed per group for ELISA (*N* = 10) and 80 follicles being analyzed per group for qPCR (*N* = 80). * *p* < 0.05, ** *p* < 0.01, and *** *p* < 0.001 vs. Control.

To assess the effect of NaBu on E_2_ production, our ELISA results showed that there was no significant difference in E_2_ levels between the NaBu and control groups on the fourth day prior to NaBu treatment. Following treatment with 0.10 mM NaBu on the fourth day, E_2_ levels increased significantly on the sixth and eighth days in the NaBu group compared with the control group (Figure 4A). Our qPCR results further showed that the mRNA levels of STAR, CYP11A1, and CYP1B1 were significantly increased in the NaBu group compared with the control group, while CYP19A1 exhibited no significant difference (Figure 4B).

### 3.5. Effects of NaBu on Follicle Viability

**Figure 5:**
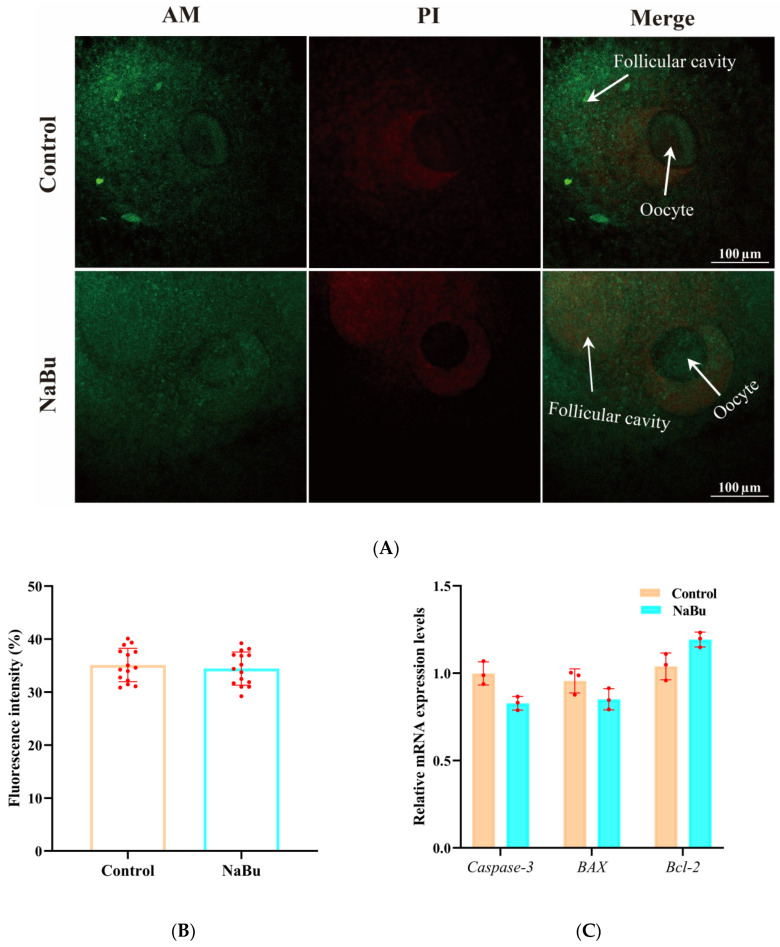
Effects of NaBu on follicular viability when added on day 4 of culture in vitro. (**A**) Representative fluorescence images of follicles stained with Calcein-AM and PI. (**B**) Quantification of PI fluorescence intensity. (**C**) Relative mRNA levels of *Caspase-3*, *BAX*, and *BCL*-2. Data represent mean ± SD from three independent experiments (*n* = 3), with 15 follicles being analyzed per group for fluorescence staining (*N* = 15) and 80 follicles being analyzed per group for qPCR (*N* = 80). Scale bar, 100 μm.

Calcein-AM and PI staining were used to evaluate follicular viability. Calcein-AM positive signals were mainly observed in ovarian granulosa cell layers, whereas there was no significant difference in the fluorescence intensity of PI between the NaBu and control groups (Figure 5A,B). Consistently, our qPCR results showed no significant differences between the mRNA levels of *Caspase-3*, *BAX*, and *BCL-2* in the NaBu and control groups (Figure 5C).

### 3.6. Effect of NaBu on F-Actin Cytoskeleton Remodeling and Distribution in the Follicles

**Figure 6:**
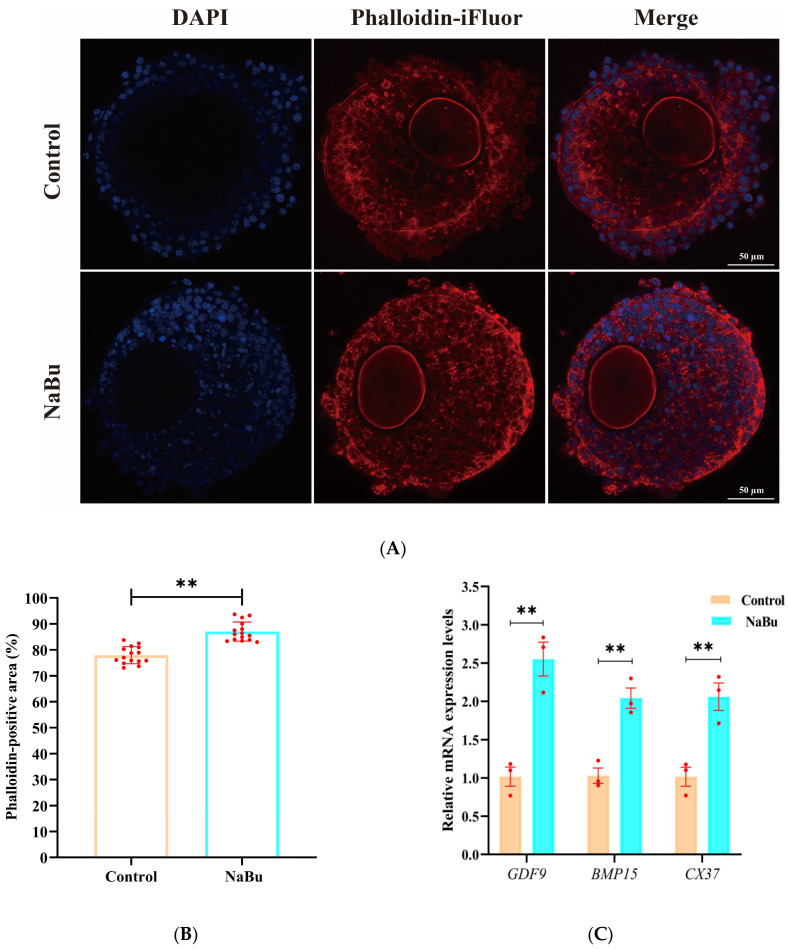
Effects of NaBu on the F-actin cytoskeleton in the follicles added on day 4 of culture in vitro. (**A**) Representative fluorescence images of follicles on the eighth day. (**B**) Quantification of the Phalloidin-positive area, reflecting the spatial extent of F-actin coverage in the follicle. (**C**) Relative mRNA levels of *GDF9*, *BMP15*, and *CX37*. Data represent mean ± SD from three independent experiments (*n* = 3), with 15 follicles being analyzed per group for fluorescence staining (*N* = 15) and 80 follicles being analyzed per group for qPCR (*N* = 80). ** *p* < 0.01 vs. Control. Scale bar, 50 μm.

F-actin microfilaments were primarily distributed between ovarian granulosa cells in a uniform network in the control group (Figure 6A). F-actin signals appeared more concentrated, and the filament arrangement was visually denser, with more filamentous extensions being observed from ovarian granulosa cells toward the oocyte in the NaBu group than in the control group (Figure 6A). The fluorescence intensity was significantly increased in the NaBu group compared with the control group (Figure 6B). Furthermore, qPCR results showed that the mRNA levels of *GDF9*, *BMP15*, and *CX37* were significantly increased in the NaBu group compared with the control group (Figure 6C).

### 3.7. Effects of NaBu on Oocyte Spindle Structure and Chromosomal Distribution

**Figure 7:**
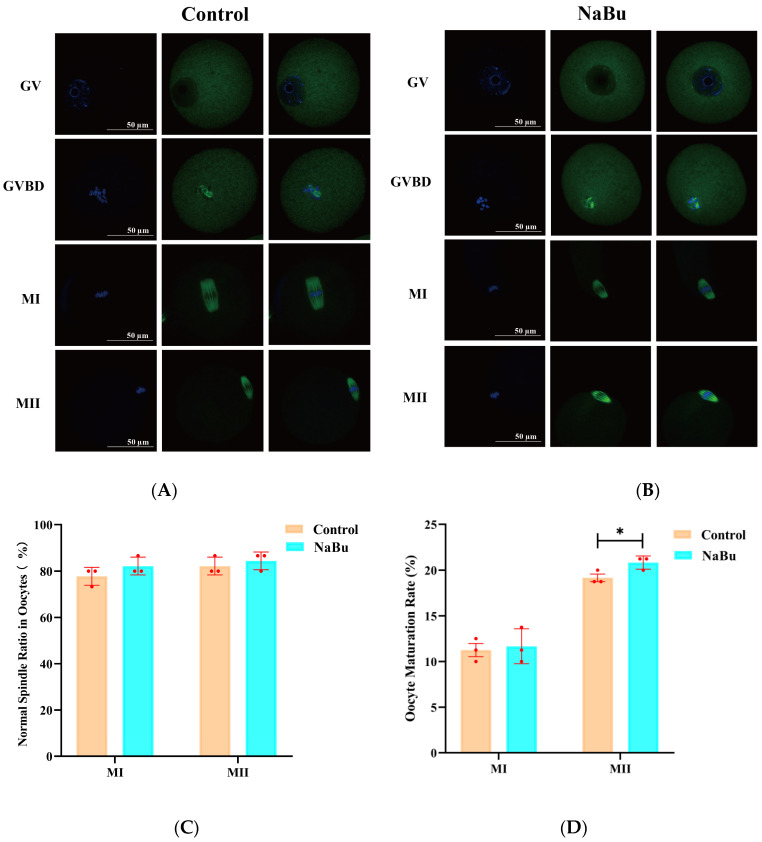
Effect of NaBu on the spindle morphology of oocytes when added on day 4 of culture in vitro. (**A**) Control group. (**B**) 0.10 mM NaBu group. (**C**) Normal spindle ratio in oocytes. (**D**) Oocyte maturation rates. Data represent mean ± SD from three independent experiments (*n* = 3), with 15 oocytes being analyzed per group for spindle morphology (*N* = 15) and 80 follicles being analyzed per group for oocyte maturation rate (*N* = 80). All images were acquired using the same microscope model (Olympus IX73) and identical magnification settings. The apparent size difference observed in the control MI images reflects the naturally larger and more centrally positioned MI spindle in the selected representative oocytes, rather than any difference in imaging magnification. * *p* < 0.05 vs. Control. Scale bar, 50 μm.

The oocytes in the GV stage exhibited dispersed chromatin with a clearly visible nucleolus and intact germinal vesicle in the control group (Figure 7A). During GVBD, the germinal vesicle began to break down, the chromosomes started to condense, and the spindle began to form, although still loosely (Figure 7A). In the MI stage, the chromosomes were aligned along the metaphase plate, with a well-formed bipolar spindle (Figure 7A). In the MII stage, the chromosomes were highly condensed and aligned, with a clearly defined polar spindle structure (Figure 7A). NaBu-treated oocytes at corresponding maturity stages exhibited overall spindle morphology and chromosomal configurations similar to the control group (Figure 7B). Quantitative analysis showed that the proportion of oocytes with normal spindle morphology at both the MI and MII stages had no significant difference between the NaBu and control groups (Figure 7C). The rate of MI-stage oocytes had no significant difference between the NaBu and control groups (Figure 7D). However, the rate of MII-stage oocytes was significantly increased in the NaBu group compared with the control group (Figure 7D).

### 3.8. Effects of NaBu on Mitochondrial Distribution and Oxidative Stress in Oocytes

**Figure 8:**
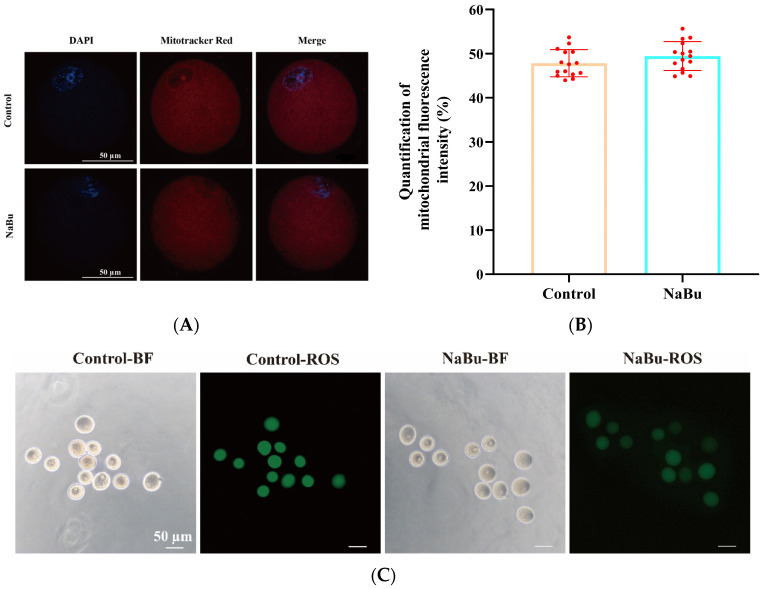

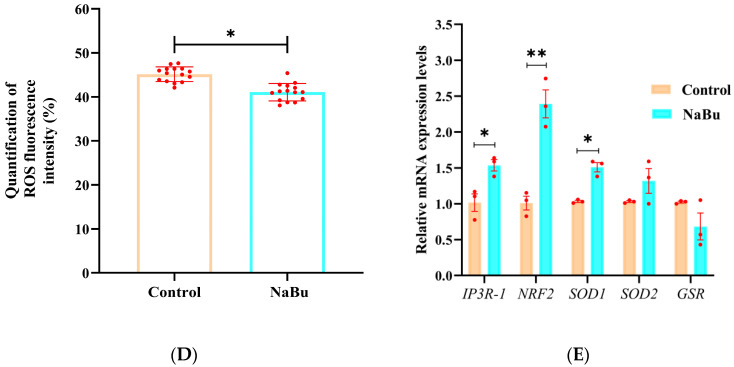
Effects of NaBu on mitochondrial function and oxidative stress in oocytes when added on day 4 of culture in vitro. (**A**) Representative fluorescence images of mitochondrial distribution in oocytes. (**B**) Quantification of mitochondrial fluorescence intensity. (**C**) Representative fluorescence images of ROS in oocytes. (**D**) Quantification of ROS fluorescence intensity. (**E**) Relative mRNA levels of *IP3R1*, *NRF2*, *SOD1*, *SOD2*, and *GSR*. Data represent mean ± SD from three independent experiments (*n* = 3), with 15 follicles being analyzed per group for fluorescence staining (*N* = 15) and 80 follicles being analyzed per group for qPCR (*N* = 80). * *p* < 0.05, and ** *p* < 0.01 vs. Control. Scale bars, 50 μm.

The oocytes exhibited evenly distributed mitochondrial fluorescence, primarily localized around the oocyte periphery, with no apparent aggregation or dispersion in the NaBu and control groups (Figure 8A). There was no significant difference in MitoTracker fluorescence intensity between the NaBu and control groups (Figure 8B).

Bright-field observations confirmed that the oocytes maintained an intact morphology and uniform cytoplasmic organization in both the NaBu and control groups (Figure 8C). However, DCFH-DA staining showed that the fluorescence intensity of ROS was significantly decreased in the NaBu group compared with the control group (Figure 8C,D).

Furthermore, our qPCR results showed that the mRNA levels of *IP3R-1*, *NRF2* and *SOD1* were significantly increased, while *SOD2* and *GSR* showed increasing trends but without significant differences between the NaBu group and the control group (Figure 8E).

### 3.9. Effects of NaBu Supplementation During Follicle Growth in 3D In Vitro Culture on the Development of Parthenogenetic Pre-Implantation Embryos

**Figure 9:**
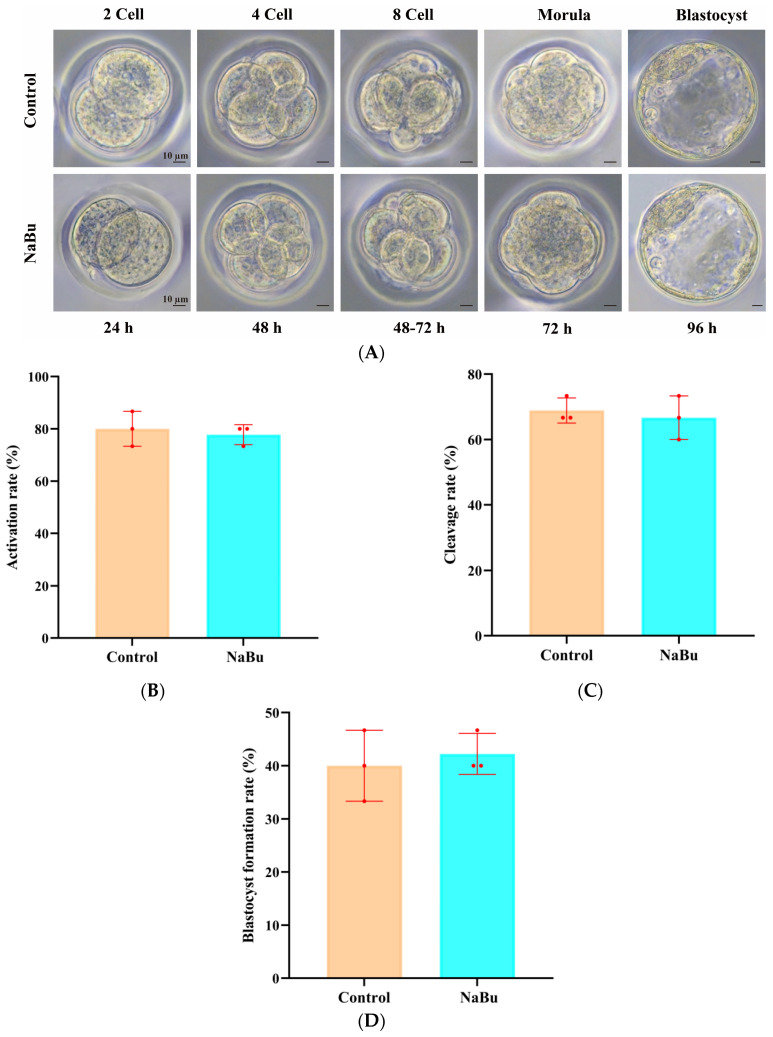
Effects of NaBu supplementation during follicle growth in 3D in vitro culture on the development of parthenogenetic embryos. (**A**) Representative morphological images of parthenogenetic embryos at different developmental stages. (**B**) Rate of activation. (**C**) Rate of cleavage. (**D**) Rate of blastocyst formation. Data represent mean ± SD from three independent experiments (*n* = 3), with 15 oocytes being analyzed per group (*N* = 15). Scale bar, 10 μm.

Parthenogenetic embryos were successfully progressed through the typical stages from two-cell to blastocyst (Figure 9A). At 24 h post-activation, the embryos had reached the two-cell stage, with symmetrical cleavage (Figure 9A). At 4872 h, the embryos had developed into four-cell and eight-cell stages, with a compact structure and minimal fragmentation (Figure 9A). At 72 h, morula-stage embryos were observed, with increased compaction (Figure 9A). At 96 h, the embryos had developed into blastocysts, with an intact zona pellucida and visible blastocoel (Figure 9A). The rate of activation was not significantly different between the NaBu and control groups (Figure 9B), which was also the case for the rates of cleavage (Figure 9C) and blastocyst formation (Figure 9D).

## 4. Discussion

NaBu, as a common feed additive, has been widely applied in the production of livestock and poultry and improves overall productive performance to a certain extent. Reproductive performance, as a key component of productive efficiency, plays a crucial role in determining the economic value of animal husbandry [15]. However, our understanding of whether NaBu can enhance reproductive performance by directly regulating the development of the reproductive system remains limited. This study utilized a 3D in vitro culture system of mouse preantral follicles to investigate the effects of NaBu on follicular development, maturation of oocytes, and early embryonic development. Our results showed that NaBu could significantly promote follicular growth, E_2_ secretion, and maturation of oocytes, while notably improving the antioxidant capacity of oocytes.

To better understand these beneficial effects, it is essential to consider how the follicles respond to NaBu exposure at different developmental stages. This study revealed a time-dependent effect of NaBu on follicular development in vitro. When NaBu was added to the culture medium on day 0 at doses of 0.05, 0.10, 0.5, and 1.0 mM, follicular diameters showed no significant differences at the beginning of culture. However, NaBu inhibited follicular growth, survival, antral formation, and ovulation in a dose-dependent manner. This phenomenon may be attributed to the physiological characteristics of the preantral follicle stage, during which the follicles have not yet formed an antrum and where their development primarily relies on local paracrine signaling between the oocytes and surrounding ovarian granulosa cells [16,17]. Oocyte-derived growth factors, such as GDF9, BMP15, and KIT ligands, play dominant roles in orchestrating the proliferation, survival, and differentiation of ovarian granulosa cells at the preantral stage, while endocrine cues and intrafollicular metabolites are less influential [18,19,20,21]. Consequently, NaBu may have a limited ability to interfere with these intrinsic pathways in the preantral follicle, explaining the lack of growth-promoting effects at the beginning of culture.

By day 4, however, the follicles had entered the early antral stage, marked by the formation of a fluid-filled antrum. This structural transition reshaped the intrafollicular microenvironment, accompanied by the accumulation of steroid hormones, proteins, and metabolites, enabling intercellular communication between ovarian granulosa cells and the oocytes [22,23]. As shown in our Supplementary Data, NaBu treatment initiated on day 0 did not promote follicular growth and instead resulted in a reduction in follicular diameter from day 4 onward. However, when NaBu was applied beginning on day 4, after the follicles had reached the early antral stage, a different pattern was observed, with NaBu-associated improvements in follicular growth and maturation of the oocytes. The formation of the antrum not only establishes a functional framework for hormone-mediated regulation but also amplifies the importance of antioxidative and metabolic support in the follicles [10,12,24]. Together, these observations indicate that the responsiveness to NaBu may vary with the developmental stage, and follicles in the early antral phase may be more capable of mounting a positive response. This stage-dependent responsiveness underscores the importance of considering follicular structural dynamics when evaluating the reproductive effects of nutritional interventions.

It is also important to distinguish the exposure pattern of NaBu being used in our in vitro follicular culture system from dietary supplementation in livestock. NaBu that is administered through feed is gradually absorbed, metabolized by intestinal epithelial cells and the liver, and enters the circulation at relatively low and fluctuating concentrations in vivo [25]. In contrast, the in vitro model exposes the isolated follicles directly to a constant NaBu dose from the beginning of culture. This direct and continuous exposure likely exceeds the physiological range that is experienced by preantral follicles in vivo and may therefore lead to inhibitory effects when treatment is initiated in the earliest preantral stage [26]. Thus, the stage-dependent effects observed in our culture system should not be interpreted as contradictory to the beneficial outcomes reported in long-term feeding studies, as the exposure route, metabolic processing, and effective intrafollicular doses differ substantially between the two conditions.

Steroid hormone secretion is one of the most critical functions of ovarian granulosa cells during folliculogenesis, as it regulates follicular growth, oocyte competence, and the establishment of a hormonally active microenvironment. In this study, we observed a significant elevation of steroid hormone secretion after NaBu treatment, accompanied by an increase in key steroidogenic genes such as *STAR*, *CYP11A1*, and *CYP1B1*, which is consistent with an increase in E_2_ levels. These findings align with previous reports of ovarian granulosa cell models, where NaBu triggers histone H3K9 acetylation to activate steroidogenesis via the PPARγ and PGC1α pathways [10], and regulates P_4_ and E_2_ secretion through cAMP-mediated signaling [27]. Interestingly, while the trends of some genes in our study (e.g., *STAR*, *CYP11A1*) were similar to those reported in ovarian granulosa cell models, others (e.g., *CYP19A1*) did not show significant differences. Such discrepancies may be attributable to the differences in experimental models. Unlike isolated ovarian granulosa cells, our system employs intact follicles, where intercellular communication between the oocytes and ovarian granulosa cells and stage-specific regulatory factors collectively modulate steroidogenic gene expressions. This context-dependent regulation highlights the importance of assessing NaBu’s effects in the physiological architecture of whole follicles, which may better reflect in vivo follicular development. Therefore, NaBu appears to modulate steroidogenesis in a context-dependent manner that relies on an intact follicular architecture.

In addition to these biological effects, it is important to consider how NaBu reaches the interior of the follicles. As a small short-chain fatty acid with high water solubility, NaBu can readily diffuse through extracellular matrices and cross the basal lamina, which remains permeable to small metabolites during follicular growth [28]. Previous studies have shown that molecules below 500 Da can pass through the follicular basement membrane and reach ovarian granulosa cells and oocytes via gap junction mediated transfer [29,30]. Given its molecular size and physicochemical properties, NaBu is likely to enter the follicular compartment through passive diffusion and subsequently influence intracellular pathways in both ovarian granulosa cells and oocytes.

It should also be noted that NaBu was dissolved in Me_2_SO (DMSO), a commonly used solvent for small molecules in biological studies. DMSO is amphiphilic and readily permeates cell membranes due to its polar sulfoxide group and nonpolar methyl groups. Its ability to facilitate solute penetration has been widely documented [31]. However, the final concentration applied in our experiments (≤0.1%) was well below levels known to affect the follicular viability, steroidogenesis, or maturation of oocytes [32]. Thus, the biological changes observed in NaBu-treated follicles are unlikely to be attributable to DMSO itself, but rather to NaBu-specific activity.

In addition to endocrine regulation, NaBu could enhance the density of F-actin filament in ovarian granulosa cells and improve cellular connections between ovarian granulosa cells and oocytes. The cytoskeleton not only provides a structural framework but also plays active roles in cholesterol trafficking, mitochondrial dynamics, and intercellular signaling [33,34,35]. Notably, studies using isolated ovarian granulosa cells or oocytes have limited capacity to capture microfilament-related outcomes, as these systems lack the intact intercellular networks that are present in whole follicles [36]. In contrast, our follicular culture model allowed us to directly observe microfilament remodeling as a functional indicator of enhanced material transport and intercellular communication. NaBu appeared to facilitate the transfer of metabolites and signaling molecules through strengthened actin-based structures, thereby promoting oocyte–granulosa cell interactions.

Furthermore, NaBu could increase the mRNA levels of *GDF9* and *BMP15*, two oocyte-secreted members of the TGF-β superfamily that are known to stimulate the proliferation of ovarian granulosa cells, suppress apoptosis, and support steroidogenesis [37,38,39,40]. The observed upregulation of *CX37*, a key connexin that is responsible for gap junction formation, further indicates improved intercellular communications between ovarian granulosa cells and oocytes [30,41]. Importantly, previous reports have highlighted the link between microfilament dynamics, spindle organization, and cytoskeletal function during oocyte maturation [42,43]. Consistent with these findings, NaBu treatment did not adversely affect the morphology of the spindle, and the oocytes at the MII stage displayed an appropriate spindle structure and chromosome alignment. These observations suggest that NaBu supports follicular development in a manner that is compatible with meiotic spindle organization and may maintain a microenvironment that is conducive to stable cytoskeletal dynamics and effective communication between oocytes and ovarian granulosa cells.

Oocyte maturation comprises two tightly coordinated processes: nuclear and cytoplasmic maturation. Nuclear maturation involves GVBD, chromatin condensation, and meiotic spindle formation, which are tightly regulated by cell-cycle related kinases such as maturation-promoting factor (MPF, CDK1-cyclin B complex) and mitogen-activated protein kinase (MAPK) signaling [44,45]. Cytoplasmic maturation, on the other hand, entails mitochondrial redistribution, ATP production, and the accumulation of maternal transcripts and proteins that are required for fertilization and early embryonic development [46,47]. This process is critically influenced by oocyte–granulosa cell communication through the cAMP-PKA signaling as well as the PI3K-AKT pathways, which supports organelle reorganization and metabolic activation [48,49,50].

In our study, NaBu increased the proportion of the oocytes reaching the MII stage significantly, and these oocytes displayed a proper spindle structure and chromosome alignment, which is consistent with effective nuclear maturation. In addition, mitochondria exhibited a uniform perinuclear distribution, suggesting coordinated cytoplasmic maturation, potentially supported by improved oxidative stress defense and metabolic conditions within the follicular microenvironment. Together, these results indicate that NaBu supports meiotic progression and maintains cellular features that are associated with both nuclear and cytoplasmic maturation.

Importantly, oxidative stress represents a major challenge in follicular culture in vitro, as excessive ROS accumulation is almost inevitable under artificial conditions. Elevated ROS levels not only compromise the viability of ovarian granulosa cells and mitochondrial homeostasis but also disrupt steroidogenesis and oocyte maturation [51,52,53]. In our study, NaBu markedly reduced intracellular ROS levels, highlighting its potential to alleviate the oxidative burden that is inherent to folliculogenesis in vitro. To our knowledge, this is the first report demonstrating an antioxidative effect of NaBu within the follicular culture system.

Notably, IP3R1 has been implicated in the regulation of oxidative stress, as its dysregulation is associated with mitochondrial dysfunction, ROS accumulation, and stress-induced apoptosis [54,55,56]. This suggests that IP3R1 may serve as a node connecting oxidative stress to follicular quality control. Previous studies have reported that NaBu can influence NRF2 signaling and modulate downstream antioxidant genes such as *SOD1* and *SOD2* [57,58]. In our study, NaBu treatment was associated with increased mRNA levels of *NRF2* and a significant elevation in *SOD1*, whereas *SOD2* showed an upward trend without reaching statistical significance. *SOD1* and *SOD2* were representative antioxidant enzymes regulated by *NRF2*. These results suggest that NaBu may partially enhance antioxidant capacity through *NRF2*-associated mechanisms, although the extent of pathway activation remains limited based on the genes examined. Previous studies have established that *NRF2* serves as a master regulator of cellular redox homeostasis and plays a pivotal role in supporting follicular development and oocyte competence by maintaining oxidative balance and stabilizing mitochondrial function [59,60,61].

Taken together, these results indicate that NaBu promotes follicular development and enhances oocyte quality by mitigating oxidative stress, preserving mitochondrial and cytoskeletal integrity, and reinforcing antioxidative signaling. It is important to note that the overall ovulation efficiency in this in vitro follicular culture system was approximately 40%, which is consistent with commonly reported values for mouse preantral follicular cultures [13]. Such systems inherently lack the full complement of endocrine cues, vascular support, and metabolic regulation that are present in vivo, leading to reduced ovulatory competence, even when the follicles achieve morphologically normal growth. Therefore, the increase in ovulation rate after NaBu treatment should be interpreted as an improvement relative to the intrinsic limitations of the model, rather than an indication that NaBu can elevate ovulatory performance to in vivo physiological levels. This contextualizes the magnitude of the NaBu-induced enhancement and highlights the constraints that are faced in preantral follicular culture systems. This work provides novel evidence for the role of NaBu in regulating redox balance in vitro and underscores the importance of NRF2 as a potential target for improving follicular culture systems [62].

In interpreting the developmental outcomes, it is also important to acknowledge the limitations of using parthenogenetic activation rather than IVF. Parthenogenesis is used to evaluate oocyte-intrinsic developmental competence by activating the oocyte, thereby avoiding the confounding effects of sperm quality, fertilization efficiency, or paternal genomic contribution [63]. For this reason, parthenogenetic activation is widely used in studies aiming to assess oocyte cytoplasmic maturation rather than fertilization capacity. However, parthenogenetic embryos lack a paternal genome and do not undergo normal imprinting patterns; their developmental trajectory therefore does not fully reflect that of biparental embryos [64]. Thus, the unchanged rates of activation, cleavage, and blastocyst observed in our study should be interpreted as indicators of intrinsic oocyte developmental potential rather than reproductive performance in a physiological fertilization context. Although IVF can provide additional information regarding fertilization-dependent competence, previous analyses showing small effect sizes suggest that IVF may not exhibit substantial differences under the current experimental conditions. Future studies incorporating IVF-based assessment may help clarify the impact of NaBu on fertilization-dependent developmental competence further.

The ability of NaBu to enhance follicular development, promote oocyte maturation, and reduce oxidative stress suggests that it may support ovarian function under suboptimal physiological or environmental conditions. This study focused on the potential of NaBu as a supplement to improve oocyte quality and follicular development within in vitro culture systems, rather than its use as a dietary supplement. Understanding the cellular pathways that are influenced by NaBu provides a mechanistic basis for optimizing in vitro follicular culture systems, with broad applications in animal reproduction and assisted reproductive technologies. Although direct extrapolation from mice to domestic species requires caution, these findings highlight the potential of NaBu in improving assisted reproductive techniques and optimizing reproductive performance in livestock and poultry.

However, despite the improvement in MII oocyte quality, the cleavage and blastocyst rates were comparable after NaBu treatment, indicating that the enhanced oocyte morphology and intracellular conditions did not fully translate into improved developmental competence in this mouse model. This observation is consistent with known limitations of in vitro follicular culture systems, in which nuclear maturation can be achieved while cytoplasmic maturation or developmental competence remains incomplete. Such discrepancies have been widely reported across mammalian species. For example, in vitro–grown bovine and porcine oocytes frequently show compromised cytoplasmic maturation or reduced embryo-forming ability, even when their morphological quality appears satisfactory. Consistent with these findings, an agarose-based 3D culture system has been shown to enhance the developmental potential of porcine preantral follicle–derived oocyte–granulosa complexes [65], whereas a recent review emphasized the persistent challenges of attaining fully competent oocytes across mammals [66]. Together, these parallels suggest that the lack of improvement in embryonic development in our study likely reflects inherent constraints of in vitro folliculogenesis and oocyte maturation rather than the absence of NaBu itself.

Future research should explore several important aspects further. First, in vivo studies in livestock and poultry are needed to validate whether the mechanisms that we identified in the mouse model translate to physiological reproductive environments. Second, evaluating NaBu in fertilization-dependent systems, including IVF and ICSI, will help determine whether the improvements in oocyte quality can be extended to embryonic developmental competence. Third, defining dose–response relationships and long-term effects of NaBu exposure will be essential for bridging the gap between short-term in vitro treatment and the dietary supplementation that is used in practical animal production. Finally, dissecting the interactions between NaBu and other metabolic or epigenetic regulators may provide further insights into how nutritional interventions shape follicular development.

Together, these directions will contribute to a more comprehensive understanding of NaBu’s role in ovarian physiology and its potential application in reproductive biotechnology and livestock production. Its application may offer broad prospects in animal reproduction and assisted reproductive technologies.

## 5. Conclusions

Based on a well-established 3D in vitro culture system of mouse preantral follicles, this study systematically elucidated the molecular mechanisms by which NaBu promotes follicular development and enhances oocyte quality. The findings demonstrate that NaBu exerts multifaceted effects by enhancing steroidogenesis, improving cytoskeletal organization, optimizing mitochondrial function, and strengthening the antioxidant defense system, thereby synergistically improving the functional status of both follicles and oocytes. Moreover, under the present treatment conditions, NaBu showed no adverse effects on meiotic spindle structure or early parthenogenetic embryo development, indicating excellent biocompatibility. These results suggest that NaBu holds great potential as a regulatory factor for optimizing in vitro follicular culture systems and improving oocyte quality.

## Figures and Tables

**Table 1:** Primer sequences of target genes for qPCR.

Gene	Gene Bank No.	Forward (5′-3′)	Reverse (5′-3′)	Product (bp)
*CAT*	NM_009804.2	AGCGACCAGATGAAGCAGTG	TCCGCTCTCTGTCAAAGTGTG	181
*SOD1*	NM_011434.2	GGGTTCCACGTCCATCAGTA	TTTCCACCTTTGCCCAAGTC	263
*SOD2*	NM_013671.3	CCAGACCTGCCTTACGACTA	TGAAGAGCGACCTGAGTTGT	170
*GSR*	NM_010344.4	GACACCTCTTCCTTCGACTACC	CACATCCAACATTCACGCAAG	142
*NRF2*	NM_010902.5	TAGATGACCATGAGTCGCTTGC	GCCAAACTTGCTCCATGTCC	153
*IP3R-1*	JQ839262.1	AGGAGAATCTCTCCCTTCTCC	GAGCCCTCTGTGCTGAAGAG	155
*STAR*	GQ415073.1	ACCAACAAAGGAGCAGCAA	TCAGGGACCTCAAAGTTCATC	103
*CYP11A1*	NM_214055.1	ACCTGGACCTTGGTTCTCTG	CATCTGCCTGATGCTCTTGT	83
*CYP19A1*	AF518322.1	CTGGCAGAAAACAACCTGAACC	TGATTCTCATCAAGCAGGTCTCC	94
*CYP1B1*	NM_001364889.1	CACTATTACGGACATCTTCGG	AGGTTGGGCTGGTCACTC	168
*GDF9*	NM_001439459.1	GATGGTGGACCTGCTGTTTA	GAGGAAGAGGCAGAGTTGTTC	99
*BMP15*	NM_009757.5	AGTGTACCTCAGCCTTCCT	GGGCAATCATACCCTCATACTC	109
*CX37*	NM_008120.3	CCCACATCCGATACTGGGTG	CGAAGACGACCGTCCTCTG	220
*BCL-2*	NM_009741.5	GATGACTGAGTACCTGAACCG	CAGAGACAGCCAGGAGAAATC	124
*BAX*	NM_007527.4	CGGCGAATTGGAGATGAACTG	GCAAAGTAGAAGAGGGCAACC	161
*Caspase-3*	NM_001284409.1	TGACTGGAAAGCCGAAACTC	GCAAGCCATCTCCTCATCAG	101
*GAPDH*	NM_001411840.1	TGTGTCCGTCGTGGATCTGA	TTGCTGTTGAAGTCGCAGGAG	150

## Data Availability

The data that support the findings of this study are available upon request.

## Supplementary Materials

The following supporting information can be downloaded at: https://www.mdpi.com/article/10.3390/ani15243567/s1, Table S1. Effect of NaBu on follicle diameter when added on day 0 of culture in vitro. Table S2. Effects of NaBu on follicle development rates added on day 0 of culture in vitro.
