# Supplementary material for: Sodium Butyrate Promotes In Vitro Development of Mouse Preantral Follicles and Improves Oocyte Quality by Regulating Steroidogenesis, Oxidative Stress, and Cytoskeleton Remodeling
Source: Animals (Basel). 2025 Dec 11;15(24):3567. doi: 10.3390/ani15243567 (PMC12730109; doi:10.3390/ani15243567)
Supplement: Supplementary file 1 [file animals-15-03567-s001.zip › animals-3973765-supplementary.pdf]

animals
MDPI

Supplementary Materials:

Sodium butyrate promotes *in vitro* development of mouse preantral follicles and improves oocyte quality by regulating steroidogenesis, oxidative stress, and cytoskeleton remodeling

Xiaohuan Liu[1†], Tuba Latif Virk[1†], Mengdie Pi[1], Qi Liu[1,2,3], Sheng Yang[1,2,3], Zhiyu Ma[1,2,3], Yuguo Yuan[1,2,3] and Fenglei Chen[1,2,3*]

[1] College of Veterinary Medicine, Yangzhou University, Yangzhou 225009, China; lxh136288@163.com (X.H. Liu); tuba.virk2@gmail.com (T. L. Virk); pp010177@163.com (M.D. Pi); qiliu@yzu.edu.cn (Q. Liu); 008220@yzu.edu.cn (S. Yang); mzy2017@yzu.edu.cn (Z.Y. Ma); yyg9776430@163.com (Y.G. Yuan); flchen@yzu.edu.cn (F.L. Chen).
[2] Jiangsu Co-innovation Center for Prevention and Control of Important Animal Infectious Diseases and Zoonoses, Yangzhou 225009, China; qiliu@yzu.edu.cn (Q. Liu); 008220@yzu.edu.cn (S. Yang); mzy2017@yzu.edu.cn (Z.Y. Ma); yyg9776430@163.com (Y.G. Yuan); flchen@yzu.edu.cn (F.L. Chen).
[3] Joint International Research Laboratory of Agriculture and Agri-Product Safety of the Ministry of Education of China, Yangzhou University, Yangzhou 225009, China; qiliu@yzu.edu.cn (Q. Liu); 008220@yzu.edu.cn (S. Yang); mzy2017@yzu.edu.cn (Z.Y. Ma); yyg9776430@163.com (Y.G. Yuan); flchen@yzu.edu.cn (F.L. Chen).
* Correspondence: flchen@yzu.edu.cn (F.L. Chen); Tel.: +86-514-8797-9030; Fax: +86-514-8797-2218.
[†] These authors contributed equally to this work.

1. Materials and Methods

1.1 Collection of mouse preantral follicles

Female ICR mice (12.5-day-old) were obtained from the Laboratory Animal Center of Yangzhou University. All procedures were approved by the Animal Protection and Ethics Committee of Yangzhou University (Approval No. 202103322). In each replicate, 3 of mice were used, yielding 6 of ovaries and about 96 of preantral follicles. Three independent biological replicates were performed in this study, 9 of 12.5-day-old female mice were used. The mice were euthanized by CO_2 overdose, and the ovaries were collected under aseptic conditions. After removed oviducts and surrounding fat, the ovaries were rinsed in L-15 medium containing 10% FBS and 1% penicillin–streptomycin. Preantral follicles (100–130 µm, 2–4 granulosa cell layers, and intact basal membrane) were isolated using a 1 mL syringe with a 0.3 × 13 RWLB needle under a stereomicroscope and selected for subsequent culture.

1.2. Preantral follicles for 3D culture in vitro

200 µL of of follicualr growth medium, which consisted of MEM-α supplemented with 0.33 mM pyruvate, 10% FBS, 1% penicillin, 1% ITS, and 0.1 IU/mL FSH, was added to each well of an ultra-low attachment 96-well plate and pre-equilibrated at 37.5°C with 5% CO_2 for 2 h. Individual preantral follicles were seeded into each well and cultured for 8 days. On the 0th day, NaBu was added at four different doses (0.05, 0.10, 0.50, and 1.00 mM) and maintained throughout the culture period, with half of the medium refreshed every 2 days to sustain a constant concentration. Follicular development was monitored and imaged under an inverted microscope (Model CKX53, Olympus, Tokyo, Japan). On the 8th day, the follicle growth medium was replaced with oocyte mature medium, consisting of follicle growth medium, 1.5 IU/mL HCG, and 10 ng/mL EGF. COCs were assessed on the 9th day.

1.3. Observation of follicular morphology and measurement of follicular diameter

On the 2nd, 4th, 6th, and 8th day, follicular morphology, survival, diameter, and antrum formation were observed and recorded under an inverted microscope (Model CKX53, Olympus, Tokyo, Japan). Follicular diameter was measured using Image J by calculating the mean of the longest and perpendicular diameters.

1.4 Evaluation of follicle survival, antrum formation, and ovulation rates

Survival was assessed by observing follicular integrity and transparency, with no granulosa cell detachment. Antral formation rate was determined by the presence of a visible antrum, and ovulation rate was assessed by the appearance of the oocytes and COCs.

2. Results

Table S1. Effect of NaBu on follicle diameter added on day 0 of culture *in vitro*

		Diameter of follicles (μm)				
**Group**	**Number**	**0 d**	**2 d**	**4 d**	**6 d**	**8 d**
Control	80	115.92 ± 8.41	140.36 ± 10.78	162.04 ± 9.15	247.81 ± 31.47	315.22 ± 37.68
0.05 mM NaBu	80	115.48 ± 8.12	135.27 ± 9.64	160.45 ± 8.33	245.62 ± 29.88	309.14 ± 38.20
0.10 mM NaBu	80	116.05 ± 7.95	134.76 ± 9.21	158.92 ± 9.11	239.25 ± 28.12	304.77 ± 35.42
0.5 mM NaBu	80	115.71 ± 8.24	133.41 ± 8.88	150.13 ± 7.92*	180.24 ± 18.56***	172.65 ± 20.47***
1.0 mM NaBu	80	116.28 ± 8.07	130.12 ± 8.65	135.42 ± 8.01**	129.73 ± 9.22***	125.14 ± 8.78***

*P < 0.05, **P < 0.01, and ***P < 0.001 *vs.* Control group

Table S2. Effects of NaBu on follicle development rates added on day 0 of culture *in vitro*

**Groups**	**Numbe**	**Survival (%)**	**Antrum (%)**	**Ovulation (%)**
Control	80	83.75 ± 1.25	50.00 ± 1.25	32.50 ± 1.25
0.05 mM NaBu	80	75.00 ± 1.25	42.50 ± 1.25	28.75 ± 1.25
0.10 mM NaBu	80	67.50 ± 1.25*	37.50 ± 1.25*	26.25 ± 1.25*
0.5 mM NaBu	80	41.25 ± 1.25***	23.75 ± 1.25**	11.67 ± 0.72***
1.0 mM NaBu	80	7.50 ± 1.25***	2.08 ± 0.72***	1.67 ± 0.72***

*P < 0.05, **P < 0.01, and ***P < 0.001 *vs.* Control group
